# Supplementary material for: Human Health Risk Assessment from Lead Exposure through Consumption of Raw Cow Milk from Free-Range Cattle Reared in the Vicinity of a Lead–Zinc Mine in Kabwe
Source: Int J Environ Res Public Health. 2022 Apr 14;19(8):4757. doi: 10.3390/ijerph19084757 (PMC9032925; doi:10.3390/ijerph19084757)
Supplement: Supplementary file 1 [file ijerph-19-04757-s001.zip › ijerph-1657198-supplementary.pdf]

Supplementary Information

Table S1: Sampling locations in the study area of Kabwe and Chongwe (reference site)

Table S2: Microwave operating conditions for milk digestion

Table S3: Summary of Graphite furnace atomic absorption spectrophotometer (GFAAS)

Table S4: Analytical performances of lead

Table S1: Sampling locations in the study area of Kabwe and Chongwe (reference area)

S/N	Region	Sampling points	Latitude	Longitude	Description
1	Kabwe	B	14° 29' 25.727"	28° 23' 23.928"	Mining area
3	Kabwe	D	14° 29' 45.200"	28° 23' 21.371"	Mining area
2	Kabwe	F	14° 27' 55.479"	28° 24' 27.828"	Mining area
4	Kabwe	G	14° 30' 44.226"	28° 24' 53.634"	Mining area
5	Kabwe	H	14° 30' 21.985"	28° 24' 17.223"	Mining area
6	Kabwe	I	14° 22' 9.7314"	28° 34' 4.152"	Mining area
7	Kabwe	J	14° 22' 26.400"	28° 33' 32.400"	Mining area
8	Kabwe	K	14° 22' 48.216"	28° 33' 45.931"	Mining area
9	Kabwe	L	14° 22' 58.079"	28° 30' 25.848"	Mining area
10	Kabwe	M	14° 22' 39.356"	28° 30' 21.854"	Mining area
11	Kabwe	N	14° 33' 35.964"	28° 30' 36.503"	Mining area
12	Kabwe	O	14° 33' 35.099"	28° 31' 21.863"	Mining area
13	Kabwe	P	14° 33' 39.599"	28° 30' 8.964"	Mining area
14	Kabwe	Q	14° 23' 36.204"	28° 24' 45.468"	Mining area
15	Kabwe	R	14° 23' 27.924"	28° 24' 56.627"	Mining area
16	Kabwe	S	14° 23' 17.879"	28° 24' 56.627"	Mining area
17	Kabwe	T	14° 23' 37.463"	28° 24' 51.408"	Mining area
18	Kabwe	U	14° 26' 57.156"	28° 22' 21.144"	Mining area
19	Kabwe	V	14° 26' 57.299"	28° 22' 20.424"	Mining area
20	Kabwe	X	14° 27' 33.660"	28° 20' 58.235"	Mining area
21	Kabwe	Y	14° 23' 36.852"	28° 24' 18.179"	Mining area
22	Chongwe	W	15° 14' 33.001"	28° 37' 43.014"	Non-mining area
23	Chongwe	Z	15° 14' 38.616"	28° 37' 41.439"	Non-mining area
24	Chongwe	ZZ	15° 15' 40.880"	28° 37' 6.7184"	Non-mining area

Table S2: Microwave operating conditions for milk digestion

Step	Ramp (min)	Time (min)	Temperature (^{0}C)
1	5	5	160
2	1	10	190
3		10	75

Table S3: Summary of Graphite furnace atomic absorption spectrophotometer (GFAAS) operating conditions

Parameter	Operating conditions
Wave length (nm)	283.3
Slit Width (nm)	1.3
Time Constant (s)	0.1
Lamp Voltage (V)	7.5
PMT Voltage (V)	298
Carrier gas /Ar (%)	99.999
Modifier (NH4PO4) (w/w)	0.5
Volume of sample (µL)	20
Volume of modifier (µL)	20
Graphite tube	PyroTubeHR
Single Mode	BKG Correction
Background correction	Zeeman effect

Table S4 : Analytical performances of lead

Parameter	Value
Certified value (µg/g)	0.0053
Measured Value (µg/g)	0.0059
Standard Error (SE)	0.014
Standard Deviation (SD)	0.040
Limit of detection (LOD = µg/g)	0.130

Limit of quantitation (LOQ = µg/g)	0.400
Slope (Sensitivity)	0.107
Intercept	0.001
Limit of decision	0.066
Linearity (working range, µg/g)	0.120
Mean recovery rate (X± SD, n= 8)	119.8
Correlation coefficient	0.999
